# Impact of donor and recipient Epstein-Barr Virus serostatus on outcomes of allogeneic hematopoietic cell transplantation: a systematic review and meta-analysis

**DOI:** 10.1007/s00277-021-04428-9

**Published:** 2021-01-25

**Authors:** Michalina Kołodziejczak, Lidia Gil, Rafael de la Camara, Jan Styczyński, on behalf of Infectious Diseases Working Party, European Society for Blood and Marrow Transplantation

**Affiliations:** 1 Department of Anesthesiology and Intensive Care, Collegium Medicum Bydgoszcz, Nicolaus Copernicus University Torun, Antoni Jurasz University Hospital No.1, Bydgoszcz, Poland; 2 Department of Hematology and Hematopoietic Cell Transplantation, Medical University, Poznan, Poland; 3 Department of Hematology, Hospital de la Princesa, Madrid, Spain; 4 Department of Pediatric Hematology and Oncology, Collegium Medicum Bydgoszcz, Nicolaus Copernicus University Torun, Antoni Jurasz University Hospital No.1, ul. Sklodowskiej-Curie 9, 85-094 Bydgoszcz, Poland

**Keywords:** Epstein-Barr virus, EBV, Hematopoietic cell transplantation, HCT, Overall survival, Non-relapse mortality, Relapse-free survival, Relapse incidence, Graft-versus-host disease, GVHD

## Abstract

Allogeneic hematopoietic cell transplant (allo-HCT) is a potentially curative therapeutic strategy that showed encouraging long-term outcomes in hematological diseases. A number of factors can influence post-transplant clinical outcomes. While Epstein-Barr virus (EBV) constitutes a trigger for development of various adverse conditions, no clinical study yet has been powered to assess the effect of EBV serostatus on the clinical outcomes in allo-HCT population. To systematically summarize and analyze the impact of donor and recipient EBV serostatus on transplant outcomes in allo-HCT recipients, meta-analyses were conducted. Selected endpoints were overall survival (OS), relapse-free survival (RFS), relapse incidence (RI), non-relapse mortality (NRM), acute graft-versus-host disease (aGVHD), chronic graft-versus-host disease (cGVHD), and *de novo* cGVHD. Three studies with 26,650 patients, transplanted for acute leukemias, lymphomas, chronic hematological malignancies, or non-malignant hematological diseases were included in the meta-analysis. In the whole population, with a total of 53,300 donors and recipients, the rate of EBV seropositivity was 85.1%, including 86.6% and 83.6% among transplant recipients and healthy donors, respectively. Donor EBV seropositivity increased the risk of cGVHD by 17%, de novo cGVHD by 14%, and aGHVD by 5%. Recipient EBV seropositivity increased the risk of cGVHD by 12%, de novo cGVHD by 17%; increased NRM by 11%, increased RI by 11%, decreased OS by 14%, and decreased RFS by 11%. In performed meta-analyses, donor and recipient EBV seropositivity was found to have a significant impact on transplant outcomes in patients after allo-HCT.

## Introduction

Epstein-Barr virus (EBV) is a widespread human herpesvirus (HHV4), infecting the majority of children, that establishes lifelong latent infection in the host memory B cells [1–3]. This virus accounts for a number of clinical syndromes and conditions, including post-transplantation lymphoproliferative disorder (PTLD), one of the most serious allogeneic hematopoietic cell transplantation (allo-HCT) complications [3, 4]. Pretransplant EBV seropositivity of recipient and donor constitutes a major trigger of the PTLD development [5], affecting a dismal survival rate after HCT (20% PTLD vs. 62% non-PLTD patients) [6]. A potential post-allo-HCT complication that might pose a diagnostic challenge to be differentiated from PLTD is graft-versus-host disease (GVHD), which, depending on the severity, guards the delicate balance between transplant-related morbidity/mortality and the risk of relapse.

As thorough, evidence-based assessment of hematological diseases require a large sample size and a sufficient follow-up, the European Society for Blood and Marrow Transplantation (EBMT) undertook an action to facilitate research outcomes by joining collaborating centers. In the series of publications, the EBMT sought to define the EBV role on transplant outcomes in selected hematological diseases and a possible impact of EBV seropositivity on acute and chronic GVHD was shown [7–9]. However, no clear impact of EBV serostatus on other transplant outcomes was unveiled so far. We therefore aimed to systematically summarize and analyze the current evidence base regarding impact of donor and recipient EBV serostatus on transplant outcomes in allo-HCT recipients based on meta-analysis.

## Methods

### Data source and literature search strategy

The meta-analysis was performed according to established methods recommended by the Cochrane guidelines [10]. The findings were reported in compliance with the PRISMA (Preferred Reporting Items for Systematic Reviews and Meta-Analyses) statement for conducting systematic reviews and meta-analyses in health care interventions [10, 11]. A systematic inquiry of publications indexed in the PubMed, MEDLINE, Cochrane Central Register of Controlled Trials, Google Scholar, and EMBASE databases, as well as clinicaltrials.gov were searched until August 2020. The search was performed using the following key words and search phrases “(Epstein-Barr virus OR Epstein-Barr virus OR EBV OR EBV serostatus) AND (graft versus host OR GVHD OR overall survival OR OS OR non-relapse mortality OR NRM OR relapse-free survival OR RFS OR relapse incidence OR RI) AND (hematopoietic stem cell transplantation OR HSCT OR hematopoietic cell transplantation OR HCT OR umbilical cord blood transplantation OR UCBT OR cord blood transplantation OR CBT OR bone marrow transplantation OR BMT).” Relevant citations were screened at the title/abstract level and retrieved as full reports. Inclusion criteria were the following: (1) human studies, (2) studies reporting clinical outcomes of interest, (3) a minimum median follow-up of 1 year, (4) studies conducted in patients with hematological disorders. Exclusion criteria were the following: (1) EBV serostatus of both donor and recipient not reported, (2) studies evaluating the treatment of EBV-related post-transplant lymphoproliferative disorders, (3) studies evaluating EBV prophylaxis.

### Study design and endpoint selection

Selected endpoints were overall survival (OS), relapse-free survival (RFS), relapse incidence (RI), non-relapse mortality (NRM), acute graft-versus-host disease (aGVHD), chronic graft-versus-host disease (cGVHD), and de novo cGVHD. RFS was defined as survival without evidence of relapse or progression. Relapse was considered as the presence of >5% bone marrow (BM) blasts and/or reappearance of the underlying disease. OS was analyzed as the time from allo-HSCT to death, regardless of the cause. Death from any cause was regarded as event for OS, while relapse and death regardless from the cause were considered to be events for RFS. RI was estimated considering relapse or reappearance of the underlying disease as event of interest and death without relapse as a competing event. NRM was defined as death with no evidence of relapse or progression, and with relapse as a competing event. AGVHD was defined according to the classical criteria [12]. CGVHD was defined as limited or extensive. De novo cGVHD was defined as cGVHD occurring without previous aGVHD. The endpoints were stratified by EBV serostatus of donor (D−, D+), recipient (R−, R+) and combined recipient/donor serostatus (R−/D−, R−/D+, R+/D−, and R+/D+).

### Data collection and quality assessment

Data were abstracted on pre-specified forms, internal validity and the potential risk of bias of the included studies (according to the Cochrane Collaboration guidelines; bias for non-RCT studies) were appraised and independently double-checked by an investigator not involved in any of the retrieved studies (MK); divergences were resolved by discussion with a second and third investigator (JS, LG).[10]

### Statistical analysis

Risk ratios (RRs) and 95% confidence intervals (CIs) were used as summary statistics. Data were presented either as event-RR (for negative outcomes, such as NRM, RI) or non-event-RR (for positive outcomes, such as survival). Heterogeneity was assessed by the Cochran’s *Q* test [13]. Statistical heterogeneity was summarized by the *I*^2^ statistic, which quantifies the percent of variation in study results that is due to heterogeneity rather than to chance [14]. Pooled RRs were calculated using fixed–effects model. A random model was additionally performed as a sensitivity analysis [15]. The statistical level of significance for the summary treatment effect estimate was a 2-tailed *p* value <0.05. Review Manager, version 5.1 (The Nordic Cochrane Centre, Copenhagen, Denmark) was used for statistical computations.

## Results

### Study selection

**Fig. 1:**
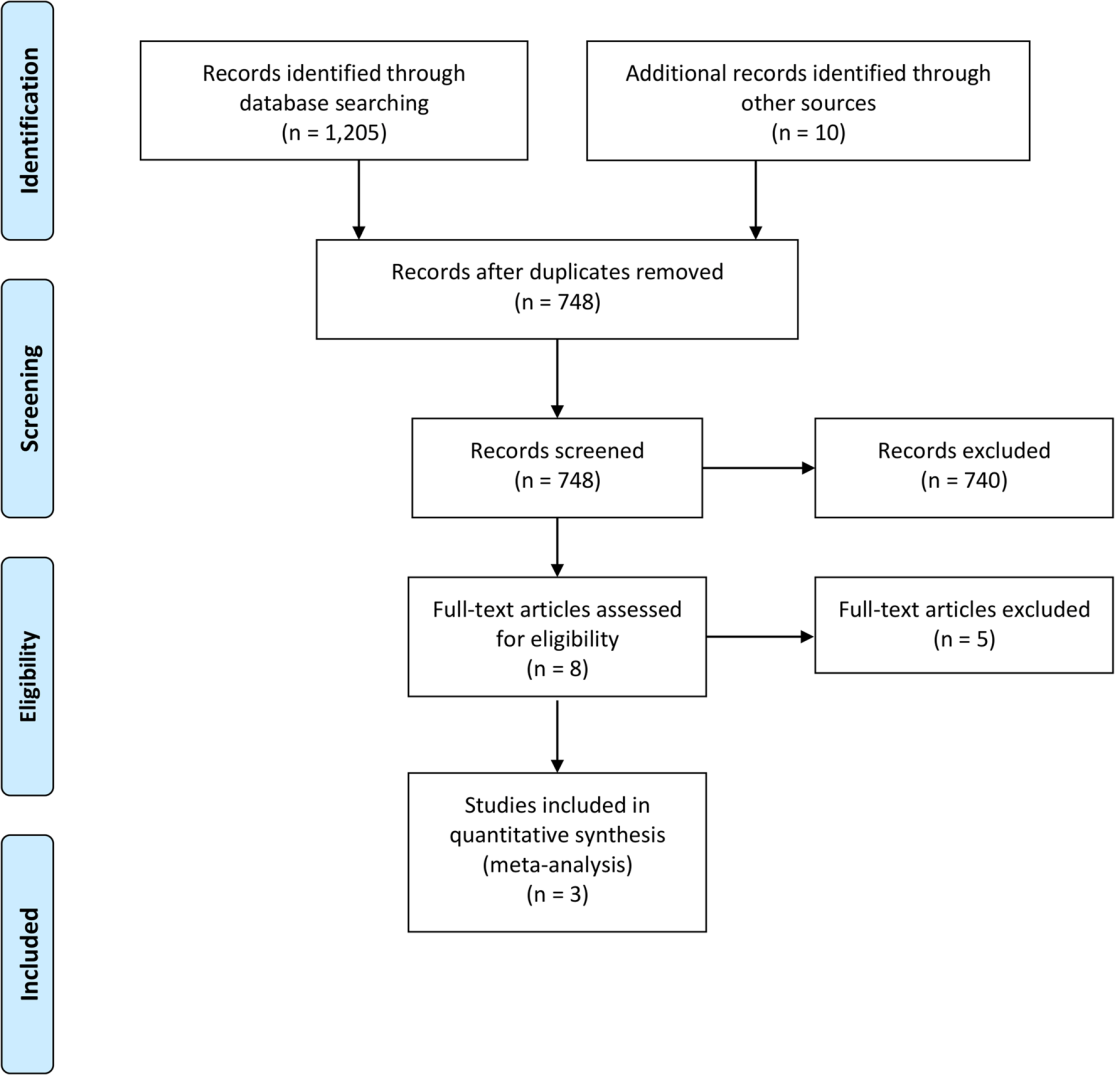
PRISMA flowchart

The PRISMA flow chart describing the publication screening process and the search strategy is depicted in Fig 1. A total of 1205 results from inquiries were identified. Additional 10 records were identified through other sources. Of the 748 potentially relevant articles, 740 were excluded based on title/abstract content; 5 studies were excluded due to unmet inclusion criteria. Three studies with 26,650 patients were included in the meta-analysis. Patients’ baseline characteristics are presented in Table 1 and the bias assessment of the included studies are listed in Table 2.

**Table 1:** Patient and transplantation characteristics

Study		Styczynski et al. 2016	Styczynski et al. 2019	Styczynski et al. 2020
Total number of patients		11,364	12,931	2355
Population type		Acute leukemia	Lymphoma or chronic malignancy	Nonmalignant hematological disorders
Transplantation type		allo-HCT	allo-HCT	allo-HCT
Male		6438 (56.7)	8216 (63.5)	1244 (52.8)
Recipient age at HCT, years*		38.9 (0.4–74.8)	51.4 (0.4–76.6)	17.3 (0.5–77.7)
Age ≥ 18 years		9277 (81.2)	12,264 (94.8)	1138 (48.3)
Underlying malignancy	Myeloproliferations/myelodysplasia	6556 (63.0)	7507 (58.1)	0
	Lymphoproliferations	3857 (37.0)	5424 (41.9)	0
	Non-malignant disorders	0	0	2355
Primary diagnosis	AML	6556 (63.0)	0	0
	ALL	3857 (37.0)	0	0
	CML	0	2175 (16.8)	0
	Chronic lymphocytic leukemia	0	1206 (9.3)	0
	MDS/MPN or MPN	0	1975 (15.3)	0
	Non-Hodgkin lymphoma	0	3327 (25.7)	0
	Hodgkin lymphoma	0	891 (6.9)	0
	Acquired bone marrow failure	0	0	1652 (70.1)
	Hemoglobinopathies	0	0	703 (29.9)
Time from diagnosis to HCT, months*		6.0 (0.1-369.7)	18.9 (0.2–527.4)	11.3 (0.1–540.7)
Status at HCT	First CR/CP	10,725 (94.4)	3654 (28.3)	NA
	Other	639 (5.6)	9277 (71.7)	NA
Sex match (recipient/donor)	Male/female	2405 (21,4)	2822 (22.1)	525 (22.5)
	Other	8824 (78.6)	9956 (77.9)	1810 (77.5)
CMV match (recipient/donor)	-/-	3639 (32.2)	4008 (31.2)	593 (25.2)
	-/+	1344 (11.9)	1670 (13.0)	238 (10.1)
	+/-	2398 (21.2)	2824 (22.0)	477 (20.3)
	+/+	3926 (34.7)	4330 (33.7)	1047 (44.5)
Source of stem cell source	PB	6593 (58.0)	9832 (76.0)	543 (23.0)
	BM	4771 (42.0)	3099 (24.0)	1706 (72.5)
	CB	0	0	106 (4.5)
Donor type	Sibling	6205 (54.7)	6111 (47.2)	1570 (66.7)
	Matched other relative	117 (1.0)	97 (0.8)	0
	Matched unrelated	1391 (12.3)	1469 (11.4)	0
	Mismatched relative	393 (3.5)	540 (4.2)	104 (4.4)
	Mismatched unrelated	654 (5.8)	553 (4.3)	0
	Unrelated	2593 (22.8)	4161 (32.2)	681 (28.9)
T cell in vivo		4701 (41.4)	7552 (58.4)	418 (17.8)
T cell ex vivo		1093 (10.1)	986 (7.9)	2205 (93.6)
Conditioning regimen	Standard (MAC)	8315 (73.2)	55,336 (41.3)	1489 (63.2)
	Reduced (RIC)	3049 (26.8)	7595 (58.7)	866 (36.8)
Year of HCT*		2008 (1997–2016)	2010 (1997–2016)	2010 (1997–2016)
Follow-up, years*		4.93 (4.8–5.0)	4.7 (4.5–4.8)	4.6 (4.4–4.8)
Donor age at HCT, years*		37.1 (0.1–81.5)	40.7 (0.0–86.0)	23.2 (0.0–76.2)

*Median (range)

*ALL*, acute lymphoblastic leukemia; *AML*, acute myeloblastic leukemia; CML, chronic myeloblastic leukemia; *MDS*, myelodysplastic syndrome; *MPN*, myeloproliferative neoplasms; *BM*, bone marrow; *PB*, peripheral blood; *CB*, cord blood; *CMV*, cytomegalovirus; *CP*, chronic phase (in CML); *CR*, complete remission; *HCT*, hematopoietic cell transplantation; *MAC*, myeloablative conditioning; *RIC*, reduced-intensity conditioning; *NA*, not available

**Table 2:** Bias assessment of the included studies

	Styczynski et al. 2016	Styczynski et al. 2019	Styczynski et al. 2020
Bias due to confounding	Low	Low	Low
Bias in selection of participants into the study	High	High	High
Bias in classification of interventions	Low	Low	Low
Bias due to deviations from intended intervention	Low	Low	Low
Bias due to missing data	Low	Low	Low
Bias in measurement of outcomes	Low	Low	Low
Bias in selection of the reported result	Low	Low	Low

### Epidemiology of EBV seropositivity

Out of 26,650 pairs of donors and recipients, the rate of EBV seropositivity among transplant recipients and healthy donors was 86.6% and 83.6%, respectively, what makes the rate of 85.1% EBV seropositivity in a population of 53,300 participants. With respect to specific subgroups: 77.1% were R+/D+, 9.4% were R+/D−, 6.5% were R−/D+, and 7.0% were R−/D−.

### Acute graft-versus-host disease

**Fig. 2:**
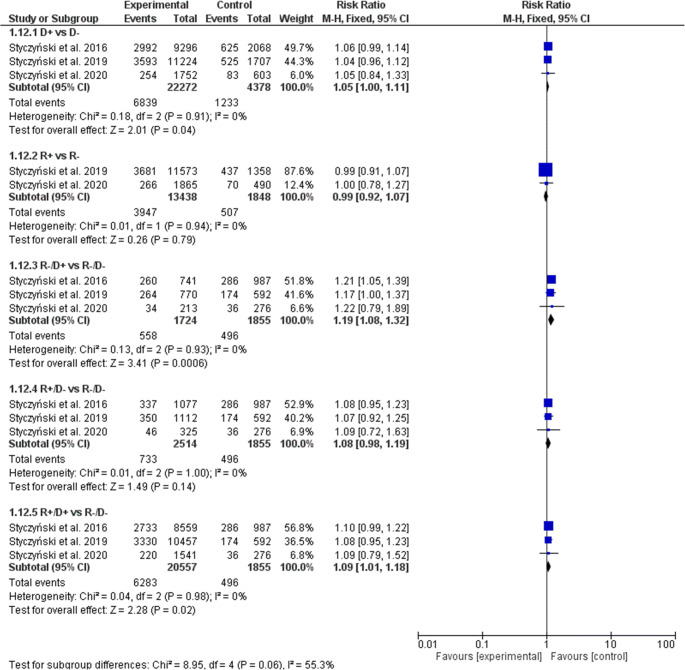
Individual and summary risk ratios with 95% CIs for the outcome of aGVHD in patients undergoing allogeneic hematopoietic cell transplantation stratified by donor and recipient EBV serostatus

A statistically significant effect of donor but not recipient (R+ vs. R− risk ratio [RR], 0.99; 95% confidence interval [CI], 0.92–1.07, *p* = 0.79) EBV serostatus on the prevalence of aGVHD was observed (Fig. 2). In the D+ serostatus arm, 6839 of 22,272 patients (30.71%) developed aGVHD compared with 1233 of 4378 (28.16%) D− patients (RR, 1.05; 95% CI, 1.00–1.11; *p* = 0.04; heterogeneity *p* = 0.91; *I*^2^ = 0%), which resulted in a statistically significant increase of aGVHD in both seronegative recipients (R−/D+ 32.37% (558 of 1724) vs. R−/D− 26.74% (496 of 1855); RR, 1.19; 95% CI, 1.08–1.32; *p* = 0.0006; heterogeneity *p* = 0.93; *I*^2^ = 0%), and seropositive recipients (R+/D+ 30.56% (6283 of 20,557) vs. R−/D− 26.74% (496 of 1855); RR, 1.09; 95% CI, 1.01–1.18; *p* = 0.02; heterogeneity p = 0.98; *I*^2^ = 0%). The significance of the estimates was not altered when random model was applied (Table 3).

**Table 3:** Comparison of results of fixed and random models of meta-analysis

			D+ vs. D−	R+ vs. R−	R−/D+ vs. R−/D−	R+/D− vs. R−/D−	R+/D+ vs. R−/D−
aGVHD	Fixed	RR	1.05 [1.00–1.11]	0.99 [0.92–1.07]	1.19 [1.08–1.32]	1.08 [0.98–1.19]	1.09 [1.01–1.18]
	Random	RR	1.05 [1.00–1.11]	0.99 [0.92–1.07]	1.19 [1.08–1.32]	1.08 [0.98–1.19]	1.09 [1.01–1.18]
	I^2^		0%	0%	0%	0%	0%
cGVHD	Fixed	RR	1.17 [1.12–1.22]	1.12 [1.05–1.19]	1.10 [1.00–1.20]	1.10 [1.01–1.20]	1.27 [1.19–1.36]
	Random	RR	1.18 [0.99–1.40]	1.07 [0.91–1.26]	1.10 [1.00–1.20]	1.09 [0.95–1.24]	1.24 [1.07–1.43]
	I^2^		92%	59%	0%	49%	71%
de novo cGVHD	Fixed	RR	1.14 [1.07–1.21]	1.17 [1.07–1.28]	1.00 [0.87–1.13]	1.09 [0.97–1.23]	1.24 [1.13–1.36]
	Random	RR	1.14 [0.99–1.31]	1.05 [0.75–1.47]	1.00 [0.88–1.14]	1.03 [0.78–1.36]	1.21 [1.06–1.39]
	*I*^2^		75%	85%	0%	77%	43%
NRM	Fixed	RR	1.03 [0.97–1.11]	1.11 [1.00–1.23]	1.01 [0.88–1.16]	1.03 [0.91–1.17]	1.06 [0.95–1.17]
	Random	RR	1.03 [0.96–1.11]	1.25 [0.85–1.83]	1.02 [0.84–1.24]	1.09 [0.82–1.46]	1.13 [0.89–1.43]
	*I*^2^		9%	76%	36%	73%	74%
OS	Fixed	RR	1.01 [0.97–1.05]	1.14 [1.06–1.22]	1.05 [0.96–1.15]	1.11 [1.02–1.20]	1.08 [1.01–1.15]
	Random	RR	1.01 [0.97–1.05]	1.13 [1.05–1.22]	1.05 [0.96–1.14]	1.15 [0.92–1.44]	1.11 [0.95–1.30]
	*I*^2^		0%	0%	0%	81%	73%
RFS	Fixed	RR	1.01 [0.97–1.04]	1.11 [1.05–1.18]	1.04 [0.96-1.12]	1.11 [1.03–1.19]	1.07 [1.01–1.13]
	Random	RR	1.00 [0.97–1.04]	1.11 [1.05–1.18]	1.04 [0.96–1.12]	1.12 [0.96–1.30]	1.08 [0.98–1.20]
	*I*^2^		0%	0%	0%	68%	54%
RI	Fixed	RR	0.98 [0.93–1.04]	1.11 [1.01–1.23]	1.06 [0.93–1.19]	1.18 [1.06–1.32]	1.08 [0.99–1.19]
	Random	RR	0.98 [0.93–1.04]	0.89 [0.50–1.59]	1.06 [0.94–1.19]	1.18 [1.01–1.37]	1.05 [0.88–1.26]
	*I*^2^		0%	84%	0%	33%	61%

*aGVHD*, acute graft-versus-host disease; *cGVHD*, chronic graft-versus-host disease; *D*, donor; *NRM*, non-relapse mortality; *OS*, overall survival; *R*, recipient; *RFS*, relapse-free survival; *RI*, relapse incidence; *RR*, risk ratio

### Chronic graft-versus-host disease

**Fig. 3:**
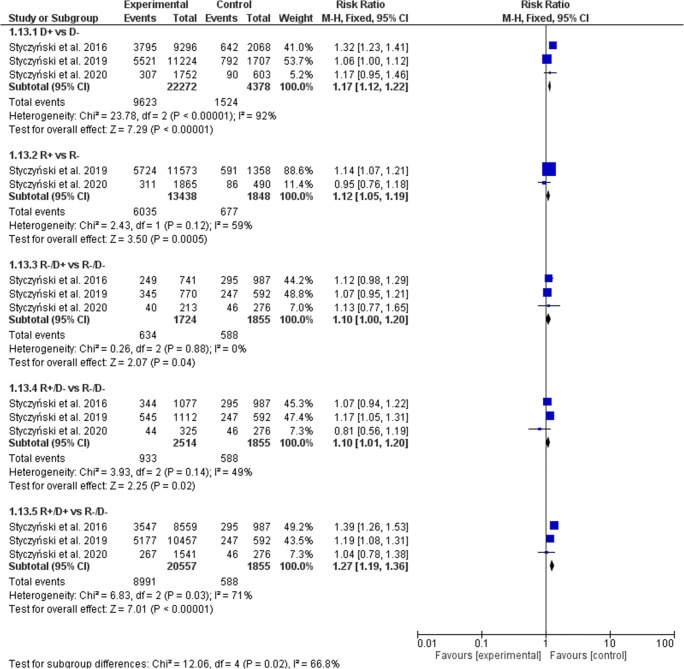
Individual and summary risk ratios with 95% CIs for the outcome of cGVHD in patients undergoing allogeneic hematopoietic cell transplantation stratified by donor and recipient EBV serostatus

The cGVHD incidence was significantly higher with both donor and recipient EBV-positive serostatus (Fig. 3). In the D+ serostatus arm, 9623 of 22,272 patients (43.21%) developed cGVHD compared with 1524 of 4378 (34.81%) D− patients (RR, 1.17; 95% CI, 1.12–1.22; *p* < 0.0001; heterogeneity *p* < 0.0001; *I*^2^ = 92%). The rate of cGVHD increased significantly with R+ patients (44.91% or 6035 of 13,438) vs. R− patients (36.63% or 677 of 1848) (RR, 1.12; 95% CI, 1.05–1.19; *p* = 0.0005; heterogeneity *p* = 0.12; *I*^2^ = 59%). In all combined subgroups, the donor and/or recipient positive EBV serostatus was associated with a significantly increased prevalence of cGVHD (R−/D+ vs. R−/D− RR, 1.10; 95% CI, 1.00–1.20; *p* = 0.04; R+/D− vs. R-/D− RR, 1.10; 95% CI, 1.01–1.20; *p* = 0.02), with the highest 1.27-fold magnitude of increase, when both donor and recipient were EBV-positive and were compared with EBV R−/D− transplants (R+/D+ 43.74% (8991 of 20,557) vs. R−/D− 31.70% (588 of 1855); RR, 1.27; 95% CI, 1.19–1.36; *p* < 0.0001; heterogeneity *p* = 0.03; *I*^2^ = 71%). When random model was applied, the increase of cGVHD did not reach statistical significance when R+ vs. R− (RR, 1.07; 95% CI, 0.91–1.26) and R+/D- vs. R−/D− (RR, 1.09; 95% CI, 0.95–1.24) were compared (Table 3).

### de novo cGVHD

**Fig. 4:**
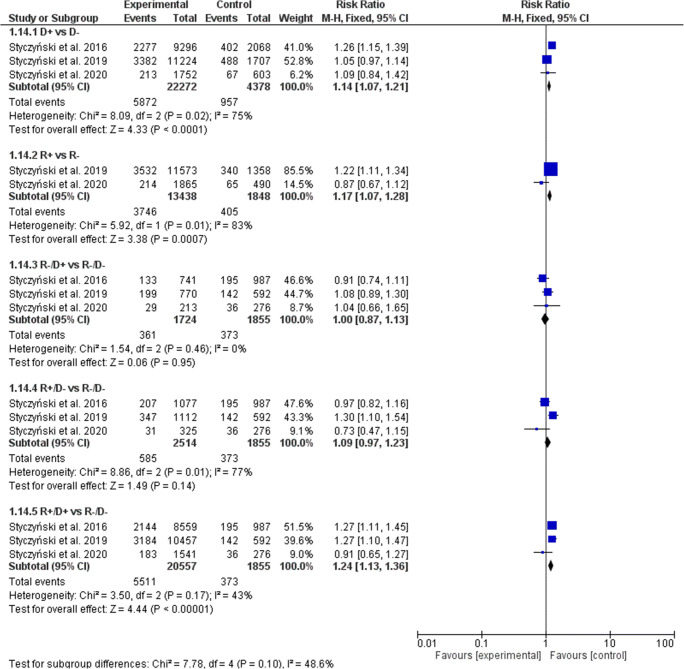
Individual and summary risk ratios with 95% CIs for the outcome of de novo cGVHD in patients undergoing allogeneic hematopoietic cell transplantation stratified by donor and recipient EBV serostatus

The de novo cGVHD significantly increased with both donor and recipient EBV-positive serostatus (Fig. 4). In the D+ serostatus arm, 5872 of 22,272 patients (26.36%) developed de novo cGVHD compared with 957 of 4378 (21.86%) D− patients (RR, 1.14; 95% CI, 1.07–1.21; *p* < 0.0001; heterogeneity *p* = 0.02; *I*^2^ = 75%). The rate of de novo cGVHD increased significantly with R+ patients (27.88% or 3746 of 13,438) vs. R− patients (21.92% or 405 of 1848) (RR, 1.17; 95% CI, 1.07–1.28; *p* = 0.0007; heterogeneity *p* = 0.01; I^2^ = 83%). In all combined donors’ or recipients’ subgroups a significantly increased prevalence of de novo cGVHD was observed only when both donor and recipient were EBV-positive and were compared with EBV R-/D- transplants (R+/D+ 26.81% (5511 of 20,557) vs. R−/D− 20.11% (373 of 1855); RR, 1.24; 95% CI, 1.13–1.36; *p* < 0.0001; heterogeneity *p* = 0.17; *I*^2^ = 43%). When random model was applied, the increase of de novo cGVHD did not reach statistical significance when R+ vs. R− (RR, 1.05; 95% CI, 0.75–1.47) and R+/D- vs. R−/D− (RR, 1.03; 95% CI, 0.78–1.36) were compared (Table 3).

### Non-relapse mortality

**Fig. 5:**
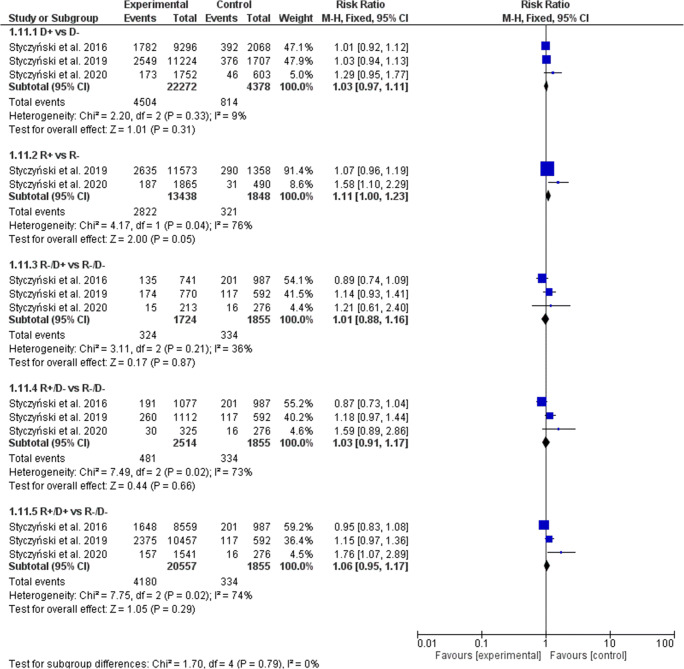
Individual and summary risk ratios with 95% CIs for the outcome of NRM in patients undergoing allogeneic hematopoietic cell transplantation stratified by donor and recipient EBV serostatus

The recipient positive EBV serostatus (21.00% or 2822 of 13,438) was associated with a numerical NRM increase compared with R- patients (17.37% or 321 of 1848) (RR, 1.11; 95% CI, 1.00–1.23; *p* = 0.05; heterogeneity *p* = 0.04; *I*^2^ = 76%) (Fig. 5).

### Overall survival

**Fig. 6:**
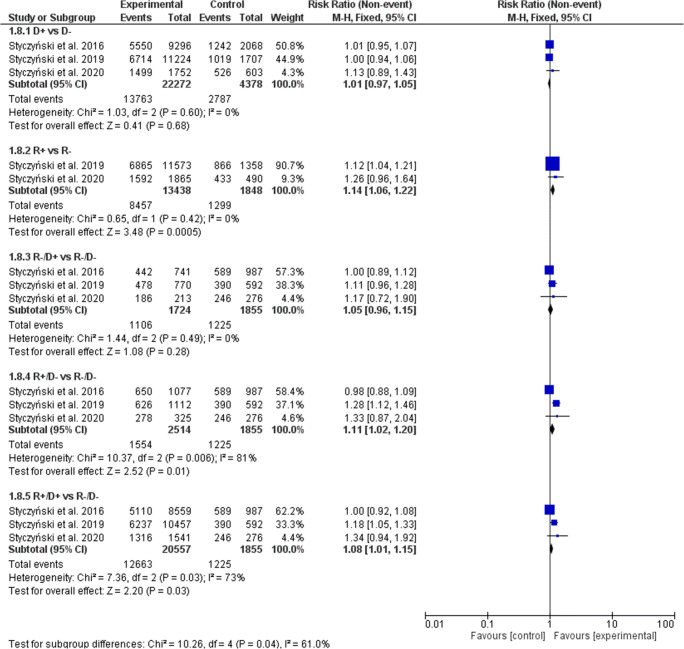
Individual and summary risk ratios with 95% CIs for the outcome of OS in patients undergoing allogeneic hematopoietic cell transplantation stratified by donor and recipient EBV serostatus

The EBV serostatus of recipients, but not donors (D+ vs. D− RR, 1.01; 95% CI, 0.97–1.05, *p* = 0.68), significantly influenced the OS (Fig. 6). In the R+ serostatus arm, 8457 of 13,438 patients (62.93%) survived compared with 1299 of 1848 (70.29%) R- patients (RR, 1.14; 95% CI, 1.06–1.22; *p* = 0.0005; heterogeneity *p* = 0.42; *I*^2^ = 0%), which resulted in a statistically significant decrease of survival of seropositive recipients when compared with seronegative recipients, regardless of donors’ serostatus (R+/D− 61.81% (1554 of 2514) vs. R−/D− 66.04% (1225 of 1855); RR, 1.11; 95% CI, 1.02–1.20; *p* = 0.01; heterogeneity *p* = 0.006; *I*^2^ = 81% and R+/D+ 61.60% (12,663 of 20,557) vs. R−/D− 66.04% (1225 of 1855); RR, 1.08; 95% CI, 1.01–1.15; *p* = 0.03; heterogeneity *p* = 0.03; *I*^2^ = 73%). The survival did not differ significantly in the R−/D+ vs. R−/D− group (RR, 1.05; 95% CI, 0.96–1.15; *p* = 0.28; heterogeneity *p* = 0.49; *I*^2^ = 0%). When random model was applied, the decrease of OS did not reach statistical significance when R+/D− vs. R−/D− (RR, 1.15; 95% CI, 0.92–1.44) and R+/D+ vs. R−/D− (RR, 1.11; 95% CI, 0.95–1.30) were compared (Table 3).

### Relapse-free survival

**Fig. 7:**
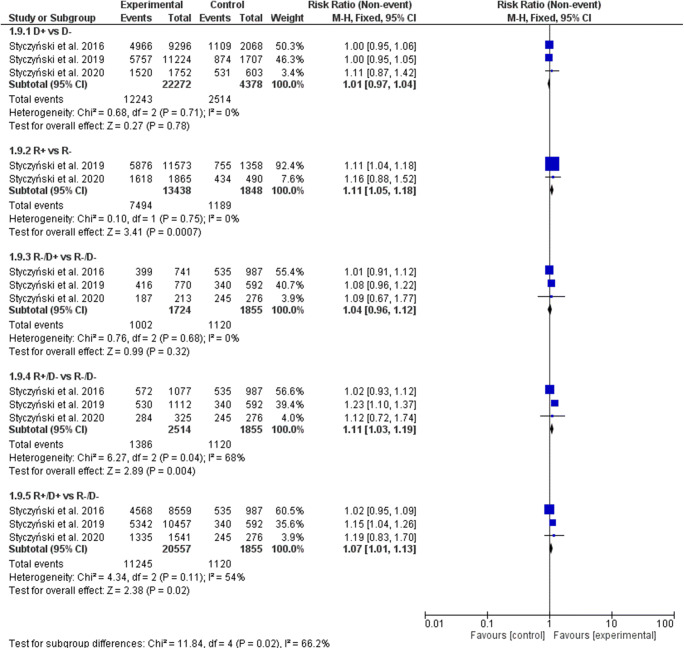
Individual and summary risk ratios with 95% CIs for the outcome of RFS in patients undergoing allogeneic hematopoietic cell transplantation stratified by donor and recipient EBV serostatus

A statistically significant effect of recipient but not donor (D+ vs. D− RR, 1.01; 95% CI, 0.97–1.04, *p* = 0.78) EBV serostatus on the RFS was observed (Fig. 7). In the R+ serostatus arm, 7494 of 13,438 patients (55.77%) survived without relapse compared with 1189 of 1848 (64.34%) R− patients (RR, 1.11; 95% CI, 1.05–1.18; *p* = 0.0007; heterogeneity *p* = 0.75; *I*^2^ = 0%), which resulted in a statistically significant decrease of RFS of seropositive recipients when compared with seronegative recipients, regardless of donor serostatus (R+/D− 55.13% (1386 of 2514) vs. R−/D− 60.38% (1120 of 1855); RR, 1.11; 95% CI, 1.03–1.19; *p* = 0.004; heterogeneity *p* = 0.04; *I*^2^ = 68% and R+/D+ 54.70% (11,245 of 20,557) vs. R−/D− 60.38% (1120 of 1855); RR, 1.07; 95% CI, 1.01–1.13; *p* = 0.02; heterogeneity *p* = 0.11; *I*^2^ = 54%). The survival did not differ significantly in the R−/D+ vs. R−/D− group (RR, 1.04; 95% CI, 0.96–1.12; *p* = 0.32; heterogeneity *p* = 0.68; *I*^2^ = 0%). When random model was applied, the decrease of RFS did not reach statistical significance when R+/D− vs. R−/D− (RR, 1.12; 95% CI, 0.96–1.30) and R+/D+ vs. R−/D− (RR, 1.08; 95% CI, 0.98–1.20) were compared (Table 3).

### Relapse incidence

**Fig. 8:**
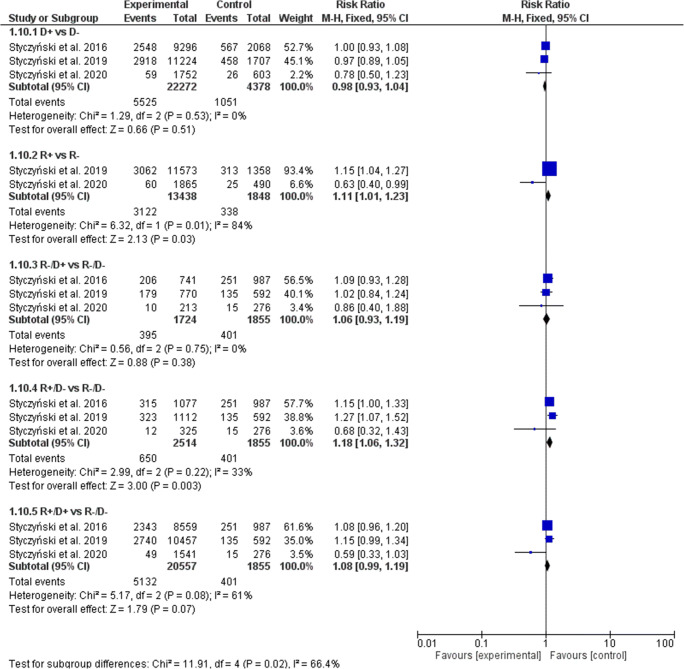
Individual and summary risk ratios with 95% CIs for the outcome of RI in patients undergoing hematopoietic allogeneic cell transplantation stratified by donor and recipient EBV serostatus

The EBV serostatus of recipients, but not donors (D+ vs. D− RR, 0.98; 95% CI, 0.93–1.04, *p* = 0.51), significantly influenced the RI rate (Fig. 8). In the R+ serostatus arm, 3122 of 13,438 patients (23.23%) relapsed compared with 338 of 1848 (18.29%) R− patients (RR, 1.11; 95% CI, 1.01–1.23; *p* = 0.03; heterogeneity *p* = 0.01; *I*^2^ = 84%), which resulted in an increase of RI of seropositive recipients when compared with seronegative recipients, regardless of donor serostatus (statistically significant with R+/D− 25.86% (650 of 2514) vs. R−/D− 21.62% (401 of 1855); RR, 1.18; 95% CI, 1.06–1.32; *p* = 0.003; heterogeneity *p* = 0.22; *I*^2^ = 33% and numerical trend with R+/D+ 24.96% (5132 of 20,557) vs. R−/D− 21.62% (401 of 1855); RR, 1.08; 95% CI, 0.99–1.19; *p* = 0.07; heterogeneity *p* = 0.08; *I*^2^ = 61%). The RI did not differ significantly in the R−/D+ vs. R−/D− group (RR, 1.06; 95% CI, 0.93–1.19; *p* = 0.38; heterogeneity *p* = 0.75; *I*^2^ = 0%). When random model was applied, the increase of RI did not reach statistical significance when R+ vs. R− (RR, 0.89; 95% CI, 0.50–1.59) and R+/D+ vs. R-/D- (RR, 1.05; 95% CI, 0.88-1.26) were compared (Table 3).

## Discussion

Our main findings are that in patients undergoing allo-HCT, positive compared with negative EBV serology is associated with (1) a statistically significant increase in development of subsequent cGVHD, de novo cGVHD, and aGVHD for both pretransplant EBV-positive donors and recipients; (2) decrease of OS, RFS, and an increase of RI in the cohort of seropositive recipients, regardless of the donor serostatus; (3) no significant, but only numerical, effect of recipient EBV serostatus on NRM.

EBV infects the majority of the population and while it remains latent in the memory B cells, the immunocompromised post-HSCT state (suppressed T cell lymphocytes allowing for the proliferation of infected B cells) can trigger its reactivation and prompt severe complications. The intensive conditioning regimens combined with baseline viral serostatus of both donor and recipient can therefore affect the transplant-related morbidity and mortality. The lack of approved complication-treatment strategies poses a challenge in the hemato-oncological management. Therefore, more detailed identification of the risk factors and assessing their impact on adverse events can provide an important insight into management strategies.

In this context, the current study focused on the impact of donor and recipient EBV seropositivity on transplant outcomes in patients after allo-HCT. Based on the studies performed within Infectious Diseases Working Party of EBMT, we hypothesized the impact of pretransplant donor and recipient EBV seropositivity on the development of GVHD. The meta-analysis of 26,650 patients with hematological malignant and non-malignant diseases undergoing allogeneic HCT showed an increase of risk of cGVHD by 17% in donor EBV seropositivity, and 12% in recipient EBV seropositivity. In specific subgroups, the risk of cGVHD was increased by 27% in R+/D+, 10% in R+/D−, and 10% in R−/D+, when compared to R−/D− transplants. The EBV serostatus was also a risk factor for development of de novo chronic GVHD in case of donor seropositivity by 14%, and recipient seropositivity by 17%. Additionally, the risk of de novo cGVHD increased by 24% in R+/D+ when compared to R−/D− transplants, but not in case of R+/D− or R−/D+ transplants. This meta-analysis has provided also an evidence of a statistically significant 5% increase of risk of aGVHD with donor but not recipient EBV seropositivity; similarly, an increase of aGVHD was found in EBV R−/D+ transplants by 19%, and in R+/D+ by 9% when compared to R−/D− transplants. These results underline the impact of donor EBV seropositivity as a risk factor for development of all types of GVHD.

Another aspect of the performed meta-analysis is a significant association between recipient, but not donor, EBV serology with other transplant outcomes. Pretransplant recipient EBV seropositivity had adverse impact on outcome decreasing OS by 14% (*p* = 0.0005) and RFS by 11% (*p* = 0.0007), while it increased NRM by 11% (*p* = 0.05) and RI by 11% (*p* = 0.03). In R+/D− subgroup, OS was decreased by 11%, and RFS was also decreased by 11%, while RI was increased by 18%, with no effect on NRM when compared to R−/D− transplants. In R+/D+ subgroup, OS was decreased by 8%, and RFS was also decreased by 7%; there was a trend towards increased RI by 8%, with no effect on NRM. While those results should be interpreted with caution, they strengthen the rationale behind the immunomodulation, anti-EBV reactivation and prolonged EBV monitoring, which duration and frequency is tailored individually. The European Conference on Infections in Leukemia guidelines advise routine EBV peripheral blood DNA surveillance, with initiation no later than 4 weeks post-HSCT and continuation at least weekly until reconstitution of cellular immunity to assert for early detection of a possible viral reactivation, PTLD diagnosis, and other clinical complications [4]. The peripheral blood EBV-DNA level is detectable even in healthy seropositive individuals, which can reflect circulating latently EBV-infected tumor cells, dying latently infected B-lymphocytes, or virions; thus, the quantification remains to be of a prominent importance. Notably, in the light of the current meta-analysis, the baseline recipient EBV serostatus (positive/negative) can be also an indicator of long-term adverse clinical outcomes and identify patients at the higher risk of long-term clinical events.

No other data are currently available on the role of EBV on transplant outcomes. Some data exist on other herpesviruses, which are known to contribute to transplant outcomes [16–18]. CMV seropositivity adversely influences overall survival [19, 20], and CMV serostatus mismatch between recipient and donor decreases overall survival in unelated-donor transplants [16]. Also, post-transplant CMV reactivation decreases survival [21]. CMV serology has been assessed as potential trigger of acute graft-versus host disease (aGVHD); however, in general, study results have been conflicted [22–24]. CMV reactivation was neither associated with subsequent development of aGVHD [21]. In contrast, the meta-analysis of the studies on the role of HHV-6B has demonstrated a strong statistical association with subsequent aGVHD [25, 26].

Our study holds several limitations and its findings should be interpreted with caution. The results of this meta-analysis were derived from study-level data and not from patient-level data, a limitation typical for this type of analysis. The results were associated with an increased heterogeneity, which might reflect a limited number of studies fulfilling the inclusion criteria. The currently available studies evaluating the EBV serostatus on allo-HCT post-transplant outcomes are still inadequate to draw definite conclusions; however, by the means of meta-analysis, we were able to derive promising estimates that can prompt the direction of further studies. We primarily used fixed model of meta-analysis, as the choice between a fixed-effect and a random-effects meta-analysis should not be made on the basis of a statistical test for heterogeneity only [10], but the results were also analyzed for comprehensiveness by a more conservative random model, which, due to the limited number of studies, awarded relatively more weight to the smaller study than it received in a fixed-effect meta-analysis. Due to the limited number of studies available additional sensitivity analyses, accounting for a potential confounders, could not be performed. The number of EBV serostatus R−/D− patients was small; however, it reflected its epidemiological prevalence in the general population.

In summary, in performed meta-analyses donor and recipient EBV seropositivity was found to have a significant impact on transplant outcomes in patients after allogeneic hematopoietic cell transplantation. More data, however, are required to provide definite conclusions on the effect of EBV seropositivity and the risk of mortality and relapse incidence.
